# Diabetic ketoacidosis in patients with SARS-CoV-2: a systematic review and meta-analysis

**DOI:** 10.1186/s13098-021-00740-6

**Published:** 2021-10-26

**Authors:** Saad Alhumaid, Abbas Al Mutair, Zainab Al Alawi, Ali A. Rabaan, Mohammed A. Alomari, Sadiq A. Al Salman, Ahmed S. Al-Alawi, Mohammed H. Al Hassan, Hesham Alhamad, Mustafa A. Al-kamees, Fawzi M. Almousa, Hani N. Mufti, Ali M. Alwesabai, Kuldeep Dhama, Jaffar A. Al-Tawfiq, Awad Al-Omari

**Affiliations:** 1 Administration of Pharmaceutical Care, Al-Ahsa Health Cluster, Ministry of Health, Rashdiah Street, P. O. Box 12944, Al-Ahsa, 31982 Saudi Arabia; 2 Research Center, Almoosa Specialist Hospital, Al-Ahsa, Saudi Arabia; 3 College of Nursing, Princess Norah Bint Abdul Rahman University, Riyadh, Saudi Arabia; 4 School of Nursing, University of Wollongong, Wollongong, Australia; 5 Division of Allergy and Immunology, College of Medicine, King Faisal University, Al-Ahsa, Saudi Arabia; 6 Molecular Diagnostic Laboratory, Johns Hopkins Aramco Healthcare, Dhahran, Saudi Arabia; 7 College of Medicine, Alfaisal University, Riyadh, 11533 Saudi Arabia; 8 Department of Public Health and Nutrition, The University of Haripur, Haripur, 22610 Pakistan; 9 Palliative Care Department, King Fahad Medical City, Riyadh, Saudi Arabia; 10 Division of Neurology, Internal Medicine Department, King Fahad Hofuf Hospital, Ministry of Health, Al-Ahsa, Saudi Arabia; 11 Administration of Nursing, Al-Ahsa Health Cluster, Ministry of Health, Al-Ahsa, Saudi Arabia; 12 Regional Medical Supply, Al-Ahsa Health Cluster, Ministry of Health, Al-Ahsa, Saudi Arabia; 13 Primary Care Medicine, Al-Ahsa Health Cluster, Ministry of Health, Al-Ahsa, Saudi Arabia; 14 Department of Pharmacy, Al Jaber Hospital for Eye, Ear, Nose and Throat, Al-Ahsa, Saudi Arabia; 15 Department of Cardiac Sciences, King Abdulaziz Medical City, Ministry of National Guard Health Affairs, Jeddah, Saudi Arabia; 16 Department Cardiac Sciences, College of Medicine, King Saud Bin Abdulaziz University for Health Sciences, Ministry of National Guard Health Affairs, Jeddah, Saudi Arabia; 17 Department of Medical Research, King Abdullah International Medical Research Center, Ministry of National Guard Health Affairs, Jeddah, Saudi Arabia; 18 Department of Restorative Dentistry, King Faisal General Hospital, Ministry of Health, Al-Ahsa, Saudi Arabia; 19 Division of Pathology, ICAR-Indian Veterinary Research Institute, Uttar Pradesh, Izatnagar, Bareilly, 243122 India; 20 Infectious Disease Unit, Specialty Internal Medicine, Johns Hopkins Aramco Healthcare, Dhahran, Saudi Arabia; 21 Infectious Disease Division, Department of Medicine, Indiana University School of Medicine, Indianapolis, IN USA; 22 Infectious Disease Division, Department of Medicine, Johns Hopkins University School of Medicine, Baltimore, MD USA; 23 College of Medicine, Alfaisal University, Riyadh, Saudi Arabia; 24 Research Center, Dr. Sulaiman Al Habib Medical Group, Riyadh, Saudi Arabia

**Keywords:** SARS-Cov-2, Diabetes, COVID-19, Ketoacidosis, Systematic Review, Meta-Analysis

## Abstract

**Background:**

One possible reason for increased mortality due to SARS-CoV-2 in patients with diabetes is from the complication of diabetic ketoacidosis (DKA).

**Objectives:**

To re-evaluate the association of SARS-CoV-2 and development of DKA and analyse the demographic and biochemical parameters and the clinical outcomes in COVID-19 patients with DKA.

**Design:**

A systematic review and meta-analysis. Preferred Reporting Items for Systematic Reviews and Meta-Analyses statement was followed.

**Methods:**

Electronic databases (Proquest, Medline, Embase, Pubmed, CINAHL, Wiley online library, Scopus and Nature) were searched from 1 December 2019 to 30 June 2021 in the English language using the following keywords alone or in combination: *COVID-19* OR *SARS-CoV-2* AND *diabetic ketoacidosis* OR *DKA* OR *ketosis* OR *ketonemia* OR *hyperglycaemic emergency* OR *hyperglycaemic crisis*. We included studies in adults and children of all ages in all healthcare settings. Binary logistic regression model was used to explore the effect of various demographic and biochemical parameters variables on patient’s final treatment outcome (survival or death).

**Results:**

Of the 484 papers that were identified, 68 articles were included in the systematic review and meta-analysis (54 case report, 10 case series, and 4 cohort studies). Studies involving 639 DKA patients with confirmed SARS-CoV-2 [46 (7.2%) were children and 334 (52.3%) were adults] were analyzed. The median or mean patient age ranged from < 1 years to 66 years across studies. Most of the patients (n = 309, 48.3%) had pre-existing type 2 diabetes mellitus. The majority of the patients were male (n = 373, 58.4%) and belonged to Hispanic (n = 156, 24.4%) and black (n = 98, 15.3%) ethnicity. The median random blood glucose level, HbA1c, pH, bicarbonate, and anion gap in all included patients at presentation were 507 mg/dl [IQR 399–638 mg/dl], 11.4% [IQR 9.9–13.5%], 7.16 [IQR 7.00–7.22], 10 mmol/l [IQR 6.9–13 mmol/l], and 24.5 mEq/l [18–29.2 mEq/l]; respectively. Mortality rate was [63/243, 25.9%], with a majority of death in patients of Hispanic ethnicity (n = 17, 27%; *p* = 0.001). The odd ratios of death were significantly high in patients with pre-existing diabetes mellitus type 2 [OR 5.24, 95% CI 2.07–15.19; *p* = 0.001], old age (≥ 60 years) [OR 3.29, 95% CI 1.38–7.91; *p* = 0.007], and male gender [OR 2.61, 95% CI 1.37–5.17; *p* = 0.004] compared to those who survived.

**Conclusion:**

DKA is not uncommon in SARS-CoV-2 patients with diabetes mellitus and results in a mortality rate of 25.9%. Mortality key determinants in DKA patients with SARS-CoV-2 infection are individuals with pre-existing diabetes mellitus type 2, older age [≥ 60 years old], male gender, BMI ≥ 30, blood glucose level > 1000 mg/dl, and anion gap ≥ 30 mEq/l.

**Supplementary Information:**

The online version contains supplementary material available at 10.1186/s13098-021-00740-6.

## Background

Diabetes is a frequent comorbidity in patients with severe acute respiratory syndrome coronavirus 2 [SARS-CoV-2], with a reported prevalence ranging from 9 to 20% [1–4]. Diabetes is also associated with more than twofold higher risk of having severe or critical corona virus disease 2019 [COVID-19] illness and about threefold increased risk of in-hospital mortality compared to SARS-CoV-2 patients without diabetes [1–4]. A possible reason for increased mortality due to SARS-CoV-2 in patients with diabetes is from the complication of diabetic ketoacidosis (DKA), one of the most serious acute complications of diabetes. DKA is characterized by the presence of hyperglycaemia [usually < 800 mg/dl and generally between 350 to 500 mg/dl], arterial pH [≤ 7.30], anion gap [> 12 mEq/l], and serum bicarbonate [≤ 15 mmol/l] [5].

In light of newer case reports, case-series and cohort studies that were done to re-evaluate the association of SARS-CoV-2 and development of DKA, we aimed to analyse the demographic and biochemical parameters and the clinical outcomes in COVID-19 patients with DKA with larger and better-quality data.

## Methods

### Design

We followed the Preferred Reporting Items for Systematic Reviews and Meta-Analyses guidelines (PRISMA) in conducting this systematic review and meta-analysis [6]. The following electronic databases were searched: PROQUEST, MEDLINE, EMBASE, PUBMED, CINAHL, WILEY ONLINE LIBRARY, SCOPUS and NATURE with Full Text. We used the following keywords: *COVID-19* OR *SARS-CoV-2* AND *diabetic ketoacidosis* OR *DKA* OR *ketosis* OR *ketonemia* OR *hyperglycaemic emergency* OR *hyperglycaemic crisis* OR *euglycemia* OR *euglycemic*. The search was limited to papers published in English between 1 December 2019 and 30 June 2021. Based on the title and abstract of each selected article, we selected those discussing and reporting occurrence of DKA in COVID-19 patients. We also utilized backward snowballing to increase the yield of potentially relevant articles (Additional file 1).

### Inclusion–exclusion criteria

We included case reports, case series and cohort studies, but excluded editorials, commentaries, case and animal studies, discussion papers, preprints, news analyses, and reviews. We considered studies to be eligible regardless of experimental or observational design, and irrespective of their primary objective. However, we excluded studies that did not report data on DKA and SARS-CoV-2; studies that never reported details on SARS-CoV-2 identified cases with DKA; or studies that reported DKA in patients with negative PCR COIVD-19 tests. We evaluated studies that included all children and adults as our population of interest who experienced DKA and SARS-CoV-2 infection during the period from December 1, 2019 through June 30, 2021.

### Data extraction

Four authors (S.A., A.A., A.R. and Z.A.) critically reviewed all of the studies retrieved and selected those judged to be the most relevant. The abstracts of all citations were examined thoroughly. Data were extracted from the relevant research studies using key headings, which are noted in Table 1, simplifying analysis, and review of the literature. Articles were categorized as case report, case series or cohort studies.

**Table 1:** Summary of the characteristics of the included studies with evidence on diabetic ketoacidosis and SARS-CoV-2 (n = 68 studies), 2019–2021

Author, year, study location	Study design, setting	Age (years)^b^	Male, n (%)	BMI (kg/m^2^)^b^	Ethnicity^a^	Type of diabetes	Use of SGLT2 inhibitors	Biochemical parameters at presentation^b^					NOS score; and Treatment outcome
								Blood glucose (mg/dl)	HbA1c (%)	pH	Bicarbonate (mmol/l)	Anion gap (mEq/l)	
Albuali et al. 2021 [8], Saudi Arabia	Retrospective case report, single centre	7	0 (0)	Not reported	1 Arab	1 Newly diagnosed	No	555	10.3	7.10	10	23	(NOS, 6) 1 survived
Alfishawy et al. 2021 [9], Egypt	Retrospective case report, single centre	17	1 (100)	Not reported	1 Arab	1 Newly diagnosed	No	566	14.7	6.8	Not reported	Not reported	(NOS, 5) 1 survived
Ali et al. 2021 [10], Qatar	Retrospective case report, single centre	53	1 (100)	Not reported	1 Bengali	1 Newly diagnosed	No	295.2	6.9	6.831	5	35	(NOS, 6) 1 died
Alizadeh et al. 2021 [11], United States	Retrospective case report, single centre	1.3	1 (100)	Not reported	Not reported	1 Newly diagnosed	No	805	9.5	7.0	4	40	(NOS, 6) 1 survived
Al-Naami et al. 2020 [12], Saudi Arabia	Retrospective case report, single centre	46	1 [100]	27	1 Arab	1 Newly diagnosed	No	657	13.5	7.4	29	26	(NOS, 5) 1 died
Alsadhan et al. 2020 [13], Saudi Arabia	Retrospective case series, single centre	47 (42–62.5)	3 (60)	29.4 (26.8–29.4)	5 Arab	2 Pre-existing T2DM 1 Pre-existing T1DM 2 Newly diagnosed	No	491 (360–664)	11.3 (10.4–14.8)	7.14 (6.97–7.27)	12.5 (8.5–14.1)	25 (19.5–26)	(NOS, 6) 4 survived 1 died
Añazco et al. 2021 [14], Peru	Retrospective case report, single centre	41	0 (0)	> 30	1 Hispanic	1 Pre-existing T2DM	No	500	Not reported	7.29	20	Not reported	(NOS, 5) 1 died
Amesfoort et al. 2021 [71], The Netherlands	Retrospective case report, single centre	21	0 (0)	Not reported	1 White (Caucasian)	Not reported	No	84.6	Not reported	7.34	8.7	23	(NOS, 6) 1 survived
Armeni et al. 2020 [15], United Kingdom	Retrospective case series, multicentre	57 [48–64]	7 (63.6)	24·7 (21·3–28·5)	5 Black 1 Asian 3 White (Caucasian) 2 Mixed	2 Pre-existing T1DM 9 Pre-existing T2DM	No	486 (396–558)	12.4 (10·7–14·2)	7.2 (6.9–7.3)	11.8 (7.8–15.4)	14.8 (10.4–20.5)	(NOS, 8) 10 survived 1 died
Batista et al. 2021 [16], Brazil	Retrospective case report, single centre	56	1 (100)	26.4	1 Hispanic	1 Pre-existing T2DM	1 Yes	118	7.2	7.28	8.9	24.1	(NOS, 6) 1 survived
Cavalcanti et al. 2020 [17], United states	Retrospective case report, single centre	23	1 (100)	Not reported	Not reported	1 Newly diagnosed	No	1384	Not reported	7.0	Not reported	Not reported	(NOS, 6) 1 died
Chamorro-Pareja et al. 2020 [18], United States	Retrospective cohort, single centre	59 (42.3–70)	32 (64)	27.15 (23.2–33)	15 Black 16 Hispanic 8 Other 3 White (Caucasian) 1 Asian 7 Unknown	6 Pre-existing T1DM 44 Pre-existing T2DM 8 Newly diagnosed	2 Yes	506 (252–1485)	HbA1c ≥ 8 (n = 30) HbA1c < 8 (n = 4) and HbA1c unknown (n = 16)	Not reported	Not reported	28.1 (14.3–41.2)	(NOS, 6) 24 survived 25 died 1 hospitalized
Chan et al. 2020 [19], United States	Retrospective case reports, single centre	50 (33.2–62)	6 (100)	24.7 (23.9–37.6)	3 Black 3 Hispanic	5 Pre-existing T2DM 1 Newly diagnosed	No	1014 (663–1116)	12.7 (11.2–13.5)	7.05 (6.83–7.21)	7.3 (5.7–9.6)	29 (27–32.2)	(NOS, 6) 2 survived 4 died
Chee et al. 2020 [20], Singapore	Retrospective case report, single centre	37	1 (100)	22.6	1 Asian	1 Newly diagnosed	No	715	14.2	7.28	12	30	(NOS, 6) 1 survived
Croft et al. 2020 [21], United States	Retrospective case reports, single centre	55 (41.5–60)	2 (40)	29.1 (20.6–33.6)	1 Black 3 Hispanic	5 Pre-existing T2DM	No	399 (284–848)	11.3 (9.6–13.4)	7.1 (7.0–7.2)	Not reported	21 (18–23)	(NOS, 6) 3 survived 1 died 1 hospitalized
Daniel et al. 2020 [22], India	Retrospective case report, single centre	15	0 (0)	19	1 Indian	1 Newly diagnosed	No	414	13.5	6.9	2	Not reported	(NOS, 5) 1 survived
Dey et al. 2021 [23], Maldives	Retrospective case reports, single centre	65.5 (53–65.5)	2 (100)	Not reported	2 Asian	2 Pre-existing T2DM	No	1084 (626–1084)	9.8 (6.6–9.8)	Not reported	Not reported	Not reported	(NOS, 5) 2 survived
Ebekozien et al. 2021 [24], United States	Retrospective cohort, multicentre	≤ 19 = (n = 30) AND > 19 = (n = 25)	23 (41.8)	> 30 (n = 9)	30 Black 15 Hispanic 10 White (Caucasian)	44 Pre-existing T1DM 11 Newly diagnosed	No	Not reported	11.1 (9–11.1)	Not reported	Not reported	Not reported	(NOS, 8) 51 survived 4 died
Emara et al. 2020 [25], Saudi Arabia	Retrospective case report, single centre	51	1 (100)	21	1 Arab	1 Pre-existing T2DM	No	592	7.8	7	Not reported	Not reported	(NOS, 5) Not reported
Ghosh et al. 2021 [26], India	Retrospective case report, single centre	60	1 (100)	Not reported	1 Indian	1 Newly diagnosed	No	540	5.1	7.20	13	18	(NOS, 6) 1 survived
Goldman et al. 2020 [27], United Kingdom	Retrospective case reports, single centre	50.5 (40.5–76.2)	Not reported	Not reported	1 White (Caucasian) 2 Asian 1 Black	3 Pre-existing T2DM 1 Newly diagnosed	1 Yes	378 (346–450)	10.8 (9.5–10.8)	7.17 (7.10–7.26)	10 (7.5–14.8)	Not reported	(NOS, 7) 1 survived 2 died 1 hospitalized
Gorthi et al. 2021 [28], United States	Retrospective case series, single centre	65 (61.5–77)	2 (40)	28.6 (24.3–31.1)	4 Black 1 White (Caucasian)	3 Pre-existing T1DM 2 Pre-existing T2DM	1 Yes	587 (370.5–723)	8.9 (8.1–10.4)	7.31 (7.11–7.33)	16 (7–18.5)	26 (20–28.5)	(NOS, 6) 4 survived 1 died
Haider et al. 2020 [29], United States	Retrospective case report, single centre	46	0 (0)	Not reported	Not reported	1 Pre-existing T1DM	No	590	Not reported	Not reported	Not reported	18	(NOS, 6) 1 survived
Hawkes et al. 2021 [30], United States	Retrospective case reports, single centre	6 [3–6]	1 (50)	Not reported	Not reported	2 Newly diagnosed	No	Not reported	Not reported	7.17 (7.1–7.17)	10.1 (10–10.1)	Not reported	(NOS, 6) 2 survived
Heaney et al. 2020 [31], United States	Retrospective case report, single centre	54	1 (100)	42.56	Not reported	1 Newly diagnosed	No	463	Not reported	7.193	9.9	31	(NOS, 6) 1 survived
Heidarpour et al. 2021 [32], Iran	Retrospective case report, single centre	36	1 (100)	Not reported	1 Persian	1 Newly diagnosed	No	500	Not reported	7	11	Not reported	(NOS, 6) 1 survived
Hollstein et al. 2020 [33], Germany	Retrospective case report, single centre	19	1 (100)	Not reported	1 White (Caucasian)	1 Newly diagnosed	No	552	16.8	7.1	Not reported	Not reported	(NOS, 6) 1 survived
Howard et al. 2021 [34], United States	Retrospective case reports, single centre	14.5 (12–14.5)	1 (50)	Not reported	Not reported	2 Newly diagnosed	No	518 (337–518)	10.9 (10.8–10.9)	6.91 (6.84–6.91)	5 (3.7–5)	27.5 (25–27.5)	(NOS, 7) 2 survived
Ishii et al. 2021 [35], Japan	Retrospective case report, single centre	33	0 (0)	Not reported	1 Asian	1 Newly diagnosed	No	638	15.7	6.74	4.8	27.2	(NOS, 6) 1 survived
Kabashneh et al. 2020 [36], United States	Retrospective case report, single centre	54	1 (100)	Not reported	Not reported	1 Pre-existing T1DM	No	1100	Not reported	6.79	4	46	(NOS, 6) 1 survived
Kaur et al. 2020 [37], United States	Retrospective case report, single centre	43	1 (100)	Not reported	Not reported	1 Pre-existing T2DM	No	948	Not reported	6.96	Not reported	27	(NOS, 6) 1 died
Kim et al. 2020 [38], South Korea	Retrospective case reports, single centre	65.5 (59–65.5)	1 (50)	Not reported	2 Asian	2 Pre-existing T2DM	No	672 (655–672)	12 (11.4–12)	7.381	18.1	Not reported	(NOS, 6) 1 died 1 hospitalized
Kuchay et al. 2020 [39], India	Retrospective case reports, single centre	34 (30–34)	3 (100)	27.3 (26.2–27.3)	3 Indian	3 Newly diagnosed	No	582 (555–582)	12 (9.6–12)	7.21 (7.07–7.21)	13 (6.1–13)	16.2 (11.9–16.2)	(NOS, 6) 3 survived
Kulick-Soper et al. 2020 [40], United States	Retrospective case report, single centre	52	0 (0)	Not reported	Not reported	1 Newly diagnosed	No	1114	17.4	7.25	Not reported	33	(NOS, 6) Not reported
Li et al. 2020 [41], China	Retrospective case reports, single centre	44 (26–44)	2 (66.7)	Not reported	3 Asian	3 Pre-existing T2DM	No	382 (298–382)	Not reported	7.22 (6.86–7.22)	Not reported	Not reported	(NOS, 6) 1 survived 2 died
Marchon et al. 2020 [42], United Kingdom	Retrospective case reports, single centre	28	0 (0)	Not reported	White (Caucasian)	1 Pre-existing T1DM	No	401.4	12.9	7.0	3.2	Not reported	(NOS, 6) 1 survived
Mondal et al. 2021 [43], India	Prospective case series, single centre	54.8 ± (11.7)	Males were > females	24.8 ± (1.92)	26 Indian	26 Pre-existing T2DM	No	Not reported	10.1 ± (1.9)	Not reported	Not reported	Not reported	(NOS, 6) 23 survived 3 died
Naguib et al. 2021 [44], United States	Retrospective case report, single centre	8	0 (0)	> 35	1 Hispanic	1 Newly diagnosed	No	429	12	7.3	14	21	(NOS, 6) 1 survived
Nielsen-Saines et al. 2021 [45], United States	Retrospective case report, single centre	7	1 (100)	16.8	1 Hispanic	1 Newly diagnosed	No	470	14.8	7.01	3.5	32	(NOS, 6) 1 survived
Omotosho et al. 2021 [46], United States	Retrospective case report, single centre	45	0 (0)	25.39	1 Hispanic	1 Pre-existing T2DM	No	344	13.7	7.22	13	18	(NOS, 6) 1 survived
Oriot et al. 2020 [47], Belgium	Retrospective case report, single centre	52	1 (100)	29	1 White (Caucasian)	1 Pre-existing T1DM	1 Yes	270	7.4	7.25	19	17	(NOS, 6) 1 hospitalized
Ozer et al. 2020 [48], Turkey	Retrospective case report, single centre	42	0 (0)	Not reported	1 White (Caucasian)	1 Pre-existing T2DM	Yes	196	Not reported	7.08	8.9	20	(NOS, 5) 1 survived
Palermo et al. 2020 [49], United States	Retrospective case reports, single centre	49 (45–49)	1 [50]	30.5 (28–30.5)	Not reported	1 Pre-existing T2DM 1 Newly diagnosed	1 Yes	395 (192–395)	10 (7.5–10)	7.21 (7.18–7.21)	17.5 (15–17.5)	Not reported	(NOS, 6) 2 survived
Panjawatanan et al. 2020 [50], United States	Retrospective case report, single centre	59	1 [100]	32	Not reported	1 Pre-existing T2DM	No	387	11.3	7.25	19	13	(NOS, 6) 1 survived
Parwanto et al. 2020 [51], Indonesia	Retrospective case report, single centre	51	1 (100)	Not reported	1 Asian	1 Pre-existing T2DM	No	369	Not reported	7.22	9.3	Not reported	(NOS, 5) 1 died
Pasquel et al. 2021 [52], United States	Retrospective cohort, multicentre	56 ± (17)	120 (57.1)	31 ± (9)	Not reported	Not reported	Not reported	523 ± (228)	11.3 ± (2.7)	Not reported	12.2 ± (4.5)	27 ± (8)	(NOS, 8) 146 survived 64 died
Pikovsky et al. 2021 [53], United Kingdom	Retrospective case reports, single centre	34 (34–34)	0 (0)	26.5 (25–26.5)	1 Asian 1 White (Caucasian)	1 Pre-existing T2DM 1 Newly diagnosed	No	77.4 (75.6–77.4)	11.5	7.0 (6.9–7.0)	6.6 (6.2–6.6)	21	(NOS, 6) 2 survived
Plasencia-Dueñas et al. 2021 [54], Peru	Retrospective case reports, single centre	64 (42.5–71.2)	3 (75)	Not reported	4 Hispanic	4 Newly diagnosed	No	740 (489–1108)	Not reported	7.17 (6.86–7.3)	11.6 (4–17.6)	Not reported	(NOS, 5) Not reported
Potier et al. 2021 [55], France	Retrospective case report, single centre	31	1 (100)	Not reported	1 White (Caucasian)	1 Newly diagnosed	No	427	Not reported	7.25	8	Not reported	(NOS, 6) 1 survived
Rabizadeh et al. 2020 [56], Iran	Retrospective case report, single centre	16	1 [100]	17.7	1 Persian	1 Newly diagnosed	No	512	12.9	6.95	8	Not reported	(NOS, 5) 1 survived
Ramos-Yataco et al. 2021 [57], Peru	Retrospective case reports, single centre	49 (33–49)	3 (100)	Not reported	3 Hispanic	3 Newly diagnosed	No	679 (625–679)	4.5	7.1 (6.6–7.1)	8 (4–8)	10	(NOS, 5) 3 survived
Ramos-Yataco et al. 2021 [58], Peru	Retrospective case series, single centre	66 (42.5–72.5)	3 (60)	Not reported	5 Hispanic	5 Pre-existing T2DM	No	538 (465.5–617.5)	5.9 (5.6–6.7)	7.2 (6.8–7.2)	7.7 (4.2–10.7)	15 (14.5–17)	(NOS, 5) 5 survived
Rao et al. 2021 (59), United States	Retrospective case series, single centre	39 (20–54)	3 (42.8)	28.6 (26.8–34)	4 White (Caucasian) 3 Hispanic	6 Pre-existing T2DM 1 Newly diagnosed	No	311 (282–596)	12.8 (10.1–13.9)	7.25 (7.18–7.37)	13 (9–19)	21 (19–33)	(NOS, 6) 6 survived 1 died
Reddy et al. 2020 (60), India	Retrospective case reports, single centre	45 (30–45)	2 (100)	Not reported	2 Indian	1 Pre-existing T2DM 1 Newly diagnosed	No	568 (555–568)	11.1 (9.6–11.1)	7.18 (7.07–7.18)	9.5 (6.1–9.5)	14 (11.9–14)	(NOS, 6) 2 survived
Shankar et al. 2021 [61], India	Retrospective case reports, single centre	13 (11–15)	3 (60)	Not reported	5 Indian	2 Pre-existing T1DM 3 Newly diagnosed	No	425 (343–513)	13.5 (11.9–15.5)	Not reported	10 (3.7–13.7)	Not reported	(NOS, 5) 5 survived
Singh et al. 2021 [63], United States	Retrospective case series, single centre	42.5 (32.2–60.2)	7 (87.5)	27.3 (24.5–39.9)	1 Black 6 Hispanic 1 Bengali	1 Pre-existing T1DM 5 Pre-existing T2DM 2 Newly diagnosed	1 Yes	454 (375–543)	11.4 (10.7–14.4)	7.15 (7.1–7.3)	12.5 (7.7–15.5)	26.5 (22.5–28)	(NOS, 6) 5 survived 3 died
Singh et al. 2021 [62], United States	Retrospective case series, single centre	47 (35–79)	7 (63.6)	25.7 (23.4–29.3)	6 Hispanic 2 Black 2 White (Caucasian) 1 Arab	8 Pre-existing T2DM 2 Newly diagnosed 1 Pre-existing T1DM	1 Yes	974 (610–1284)	13.8 (11.8–15.5)	7.01 (6.9–7.2)	5 (4–10)	34 (30–37)	(NOS, 7) 4 survived 7 died
Singh et al. 2021 [64], United States	Retrospective case report, single centre	24	1 (100)	32.1	Not reported	1 Pre-existing T1DM	No	507	15.8	7.16	2	30.6	(NOS, 6) 1 died
Smati et al. 2020 [65], France	Retrospective case report, single centre	36	0 (0)	35.2	1 Black	1 Gestational diabetes	No	111	6.1	7.22	5.8	Not reported	(NOS, 6) 1 survived
Soliman et al. 2020 [66], Qatar	Retrospective case report, single centre	0.7	Not reported	-	1 Arab	1 Newly diagnosed	No	571	8.5	7.08	7	18	(NOS, 6) 1 survived
Stack et al. 2020 [67], United States	Retrospective case report, single centre	40	1 (100)	Not reported	Not reported	1 Pre-existing T1DM	No	328	11.5	Not reported	18	20	(NOS, 6) 1 survived
Stevens et al. 2021 [68], United States	Retrospective cohort, multicentre	63.6 ± (14.2)	108 (68.8)	< 18.5 (4.5%); 18.5 < 25.0 (29.3%); 25.0 < 30.0 (30.6%); > 30.0 (29.3%)	84 Hispanic 35 Black	156 Pre-existing T2DM 1 Pre-existing T1DM	Not reported	> 250 (n = 124)	10.7 ± (2.8)	Not reported	Not reported	Not reported	(NOS, 6) 99 survived 58 died
Suwanwongse et al. 2021 [69], United States	Retrospective case reports, single centre	51 (18–51)	2 (66.7)	33 (32–33)	Not reported	3 Newly diagnosed	No	496 (353–496)	11.4 (10.4–11.4)	7.1 (7.12–7.3)	17 (15–17)	25 (19–25)	(NOS, 6) 3 survived
Thorne et al. 2021 [70], United Kingdom	Retrospective case series, single centre	31 (25.5–39.5)	0 (0)	32.5 (29.7–39)	Not reported	4 Newly diagnosed	No	Not reported	Not reported	7.4 (7.22–7.45)	14.5 (8.1–16.2)	Not reported	(NOS, 6) 4 survived
Vasconez et al. 2020 [72], United States	Retrospective case report, single centre	16	0 (0)	Not reported	Not reported	1 Pre-existing T1DM	No	687	13.5	6.77	3	21	(NOS, 6) 1 survived
Wallett et al. 2021 [73], United Kingdom	Retrospective case series, single centre	Not reported	Not reported	Not reported	White (Caucasian)	5 Pre-existing T1DM 15 Pre-existing T2DM	Not reported	465.3	Not reported	7.15	11.4	Not reported	(NOS, 5) Not reported
Xu and Zia 2020 [74], United States	Retrospective case report, single centre	55	1 (100)	Not reported	Not reported	1 Pre-existing T2DM	1 Yes	525	Not reported	7.11	8	31	(NOS, 6) 1 survived
Zavaleta et al. 2020 [75], Peru	Retrospective case reports, single centre	64 (42.5–71.2)	3 (75)	Not reported	4 Hispanic	1 Newly diagnosed 3 Unknown diabetes type	No	740 (641–1108)	14.3 (12–14.3)	7.17 (6.86–7.3)	11.6 (4–17.6)	Not reported	(NOS, 6) 2 survived 2 died

*DKA* Diabetic ketoacidosis, *SGLT2* Sodium-glucose Cotransporter-2, *SARS-CoV-2* severe acute respiratory syndrome coronavirus 2; *NOS* Newcastle Ottawa Scale, *T1DM* type 1 diabetes mellitus, *T2DM* type 2 diabetes mellitus

^a^Patients with black ethnicity include African-American, Black African, African and Afro-Caribbean patients

^b^Data are presented as median (25th-75th percentiles), or mean ± (SD)

The following data were extracted from selected studies: authors; publication year; study location; study design and setting; age; proportion of male patients; patient body mass index [BMI] and ethnicity; type of diabetes [newly diagnosed or pre-existing]; use of sodium-glucose transport protein 2 [SGLT2] inhibitors; patient biochemical parameters at hospital presentation [blood glucose level, HbA1c, pH, bicarbonate, and anion gap]; assessment of study risk of bias; and treatment outcome [survived or died].

### Quality assessment

The quality assessment of the studies was undertaken based on the Newcastle–Ottawa Scale (NOS) to assess the quality of the selected studies [7]. This assessment scale has two different tools for evaluating case–control and cohort studies. Each tool measures quality in the three parameters of selection, comparability, and exposure/ outcome, and allocates a maximum of 4, 2, and 3 points, respectively. High-quality studies are scored greater than 7 on this scale, and moderate-quality studies, between 5 and 7 [7]. Quality assessment was performed by five authors (A.S.A., M.A.A., S.A.A., M.H.A., and H.A.) independently, with any disagreement to be resolved by consensus.

### Data analysis

Descriptive statistics were used to describe the data. For continuous variables, mean and standard deviation were used to summarize the data; and for categorical variables, frequencies and percentages were reported. Differences between the COVID-19 and DKA survival group and COVID-19 and DKA death group were analyzed using the Chi-square (*χ*^*2*^) tests (or Fisher's exact tests for expected cell count < 5 in more than 20% of the cells).

To explore the effect of various demographic and biochemical parameters variables on patient’s final treatment outcome [survival or death] in COVID-19 cases who presented with DKA and included in our review, binary logistic regression model with the univariate and multivariate logistic regression of the complete model; and their odd ratios [ORs], confidence intervals (CIs) and *p*-values were produced; and forest plots were generated for visualization purposes. All *p*-values were based on two-sided tests and significance was set at a *p*-value less than 0.05. R version 4.1.0 with the packages *finalfit* and *forestplot* was used for all statistical analyses.

## Results

### Study characteristics and quality

**Fig. 1:**
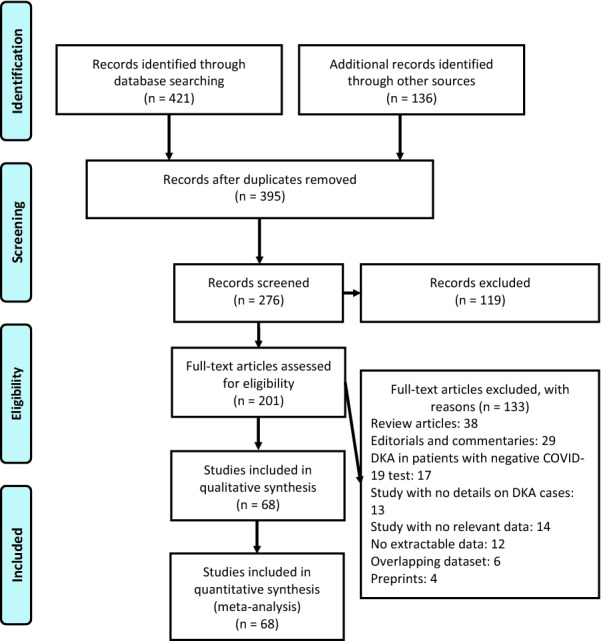
Flow diagram of literature search and data extraction from of studies included in the systematic review and meta-analysis

A total of 557 publications were identified (Fig. 1). After scanning titles and abstracts, we discarded 162 duplicate articles. Another 119 irrelevant articles were excluded based on the titles and abstracts. The full texts of the 201 remaining articles were reviewed, and 133 irrelevant articles were excluded. As a result, we identified 68 studies that met our inclusion criteria [8–75]. The detailed characteristics of the included studies are shown in Table 1. Among the included studies, 11 reported DKA and COVID-19 in children [8, 11, 22, 30, 34, 44, 45, 56, 61, 66, 72], 56 reported DKA and COVID-19 in adults [9, 10, 12–21, 23, 25–29, 31–33, 35–43, 46–55, 57–60, 62–65, 67–71, 73–75], and only 1 study reported DKA and COVID-19 in both children and adults [24]. There were 54 case report [8–12, 14, 16, 17, 19–23, 25–27, 29–42, 44–51, 53–58, 60, 61, 64, 66, 67, 69, 71, 72, 74, 75], 10 case series [13, 15, 28, 43, 58, 59, 62, 63, 70, 73] and 4 cohort [18, 24, 52, 68] studies. These studies were conducted in United States (n = 29), United Kingdom (n = 6), India (n = 6), Peru (n = 5), Saudi Arabia (n = 4), France (n = 2), Qatar (n = 2), Iran (n = 2), The Netherlands (n = 1), Turkey (n = 1), Brazil (n = 1), Belgium (n = 1), South Korea (n = 1), Japan (n = 1), Germany (n = 1), Singapore (n = 1), Indonesia (n = 1), Maldives (n = 1), China (n = 1), and Egypt (n = 1). Only 4 studies were performed within a multi-centre settings [15, 24, 52, 68]. The median NOS score for these studies was 6 (range, 5–8). Among the 68 included studies, 65 studies were moderate-quality studies (i.e., NOS scores were between 5 and 7) and 3 studies demonstrated a relatively high quality (i.e., NOS scores > 7); Table 1 (Additional file 2).

### Demographic and clinical characteristics of DKA patients with SARS-CoV-2 infection

The included studies had a total of 639 DKA patients with confirmed SARS-CoV-2 infection as detailed in Table 1. Amongst these 639 patients, 46 (7.2%) were children and 334 (52.3%) were adults. The median or mean patient age ranged from < 1 years to 66 years across studies. There was an increased male predominance in DKA patients diagnosed with SARS-CoV-2 in most of the studies [n = 373, 58.4%] [9–13, 15–17, 19, 20, 23, 25, 26, 31–33, 36, 37, 39, 45, 47, 50–52, 55–58, 60–64, 67–69, 74, 75] and majority of the patients belonged to Hispanic (n = 156, 24.4%) and black (n = 98, 15.3%) ethnicity [14–16, 18, 19, 21, 24, 27, 28, 44–46, 54, 57, 58, 62, 63, 65, 68, 75]. The median BMI for all included patients was 27.3 kg/m^2^ [interquartile range (IQR) 24.8–30.6 kg/m^2^]. Most of the patients (n = 309, 48.3%) had pre-existing type 2 diabetes mellitus, however, some of the cases were pre-existing type 1 diabetes mellitus (n = 73, 11.4%) and about (n = 75, 11.7%) of the patients were newly diagnosed diabetes mellitus with SARS-CoV-2. Only 11 (1.7%) of all cases were taking SGLT2 inhibitors.

### Biochemical parameters at presentation

The median random blood glucose level, HbA1c, pH, bicarbonate, and anion gap in all included patients at presentation were 507 mg/dl [IQR 399–638 mg/dl], 11.4% [IQR 9.9–13.5%], 7.16 [IQR 7.00–7.22], 10 mmol/l [IQR 6.9–13 mmol/l], and 24.5 mEq/l [18–29.2 mEq/l]; respectively. Five patients had blood glucose < 250 mg/dl at presentation (euglycemic DKA) [16, 53, 65, 71]; one was on SGLT2 inhibitor medication [16] while seven patients had gestational diabetes mellitus [53, 65, 70].

### Patient clinical outcome and predictors of mortality

Patients were stratified based on treatment outcome (if survived or died). A summary of the demographic, biochemical and clinical predictors with regards to final treatment outcome in 243 patients who had either survived (n = 180) or died (n = 63) is shown in Table 2. Most patients had an age of < 60 years old (n = 95, 39.1%)]. Majority of the patients were male (n = 134, 55.1%); and most of the cases belonged to Hispanic (n = 53, 21.8%) and black ethnicity (n = 45, 18.5%). There was a high obesity rate [BMI ≥ 30: n = 27, 11.1%]. Diabetes types among those patients were approximately identical [newly diagnosed (n = 61, 25.1%); pre-existing diabetes mellitus type 1 (n = 60, 24.7%); and pre-existing diabetes mellitus type 2 (n = 60, 24.7%)]. Most patients presented with a random blood glucose level in the range of 500 mg/dl and 1000 mg/dl [n = 61, 25.1%]. About 69 (28.4%) of the patients had an HbA1c higher than ≥ 10%. As expected with the acute DKA complication, most patients had abnormal arterial pH [pH between 7–7.34: n = 78, 32.1%; and pH < 7.00: n = 29, 11.9%]. Also, most patients had low bicarbonate [≤ 11 mmol/l: n = 69, 28.4%] and high anion gap [between 21–30 mEq/l: n = 39, 16%; and between 31–50 mEq/l: n = 20, 8.2%]; Table 2.

**Table 2:** Demographic data of the SARS-CoV-2 patients with diabetic ketoacidosis, stratified by treatment outcome (n = 68 studies), 2019–2021

Variable	Findings^b^			
	All (n = 243)	Survived (n = 180)	Died (n = 63)	*p-*value^c^
Age (years)				
< 60	95 (39.1)	80 (44.4)	15 (23.8)	0.021*
≥ 60	35 (14.4)	17 (9.4)	18 (28.6)	
Gender				
Female	95 (39.1)	80 (44.4)	15 (23.8)	0.015*
Male	134 (55.1)	90 (50)	44 (69.8)	
BMI (kg/m^2^)				
< 30	44 (18.1)	32 (17.8)	12 (19)	0.338
≥ 30	27 (11.1)	17 (9.4)	10 (15.9)	
Ethnicity				
Arab	10 (4.1)	7 (3.9)	3 (4.8)	0.001*
Asian	13 (5.3)	8 (4.4)	5 (7.9)	
Black^a^	45 (18.5)	42 (23.3)	3 (4.8)	
Hispanic	53 (21.8)	36 (20)	17 (27)	
Indian	14 (5.8)	13 (7.2)	1 (1.6)	
Bengali	2 (0.8)	1 (0.5)	1 (1.6)	
Persian	2 (0.8)	2 (1)	0	
White (Caucasian)	27 (11.1)	24 (13.3)	3 (4.8)	
Diabetes type				
Newly diagnosed	61 (25.1)	55 (30.5)	6 (9.5)	0.000*
Pre-existing type 1 diabetes mellitus	60 (24.7)	55 (30.5)	5 (7.9)	
Pre-existing type 2 diabetes mellitus	60 (24.7)	42 (23.3)	24 (38.1)	
Use of SGLT2 inhibitors				
Yes	8 (3.3)	6 (3.3)	2 (3.2)	0.000*
No	185 (76.1)	149 (82.8)	36 (57.1)	
Blood glucose				
< 500 mg/dl	45 (18.5)	38 (21.1)	7 (11.1)	0.048*
Between 500–1000 mg/dl	61 (25.1)	44 (24.4)	17 (27)	
> 1000 mg/dl	13 (5.3)	6 (3.3)	7 (11.1)	
HbA1c (%)				
< 10	24 (9.9)	17 (9.4)	6 (9.5)	0.096
≥ 10	69 (28.4)	57 (31.7)	12 (19)	
pH				
> 7.35	8 (3.3)	7 (3.9)	1 (1.6)	0.047*
Between 7–7.34	78 (32.1)	58 (32.2)	20 (31.7)	
< 7	29 (11.9)	18 (10)	11 (17.5)	
Bicarbonate (mmol/l)				
Above 20	3 (1.2)	2 (1.1)	1 (1.6)	0.818
Between 12–20	40 (16.5)	29 (16.1)	11 (17.5)	
Between 2–11	69 (28.4)	54 (30)	15 (23.8)	
Anion gap (mEq/l)				
Between 12–20	26 (10.7)	22 (12.2)	4 (6.3)	0.327
Between 21–30	39 (16)	29 (16.1)	10 (15.9)	
Between 31–50	20 (8.2)	12 (6.7)	8 (12.7)	

*SARS-CoV-2* severe acute respiratory syndrome coronavirus 2, *SGLT2* Sodium-glucose Cotransporter-2, *BMI* body mass index

^a^Patients with black ethnicity include African-American, Black African, African and Afro-Caribbean patients

^b^Data are presented as number (%)

^c^Chi-square (*χ*^*2*^) test was used to compare between survival and death groups

Those patients who died were more likely to have been older in age [≥ 60 years old: 28.6% vs 23.8%; *p* = 0.021]; and more likely to be men [male gender: 69.8% vs 23.8%; *p* = 0.015]. Majority of patients who died had a Hispanic ethnicity (n = 17, 27%; *p* = 0.001). Patients with a pre-existing type 2 diabetes mellitus type had the highest mortality rate compared to other diabetes types [n = 24, 38.1%; *p* = 0.000]. In addition, patients who died had higher random blood glucose level at admission [(blood glucose between 500–1000 mg/dl: 27% vs 24.4%) and (blood glucose > 1000 mg/dl: 11.1% vs 3.3%); *p* = 0.048]; and experienced more severely low pH than those who survived [pH < 7: 17.5% vs 10%; *p* = 0.047]. Moreover, more patients had high anion gap in the mortality group [anion gap between 31–50 mEq/l: 12.7% vs 6.7%, *p* = 0.327]. However, a higher proportion of patients had low bicarbonate [bicarbonate between 2–11 mmol/l: 23.8% vs 30%; *p* = 0.818] and glycated haemoglobin was raised more in the survival group [HbA1c ≥ 10%: 19% vs 31.7%; *p* = 0.096].

**Fig. 2:**
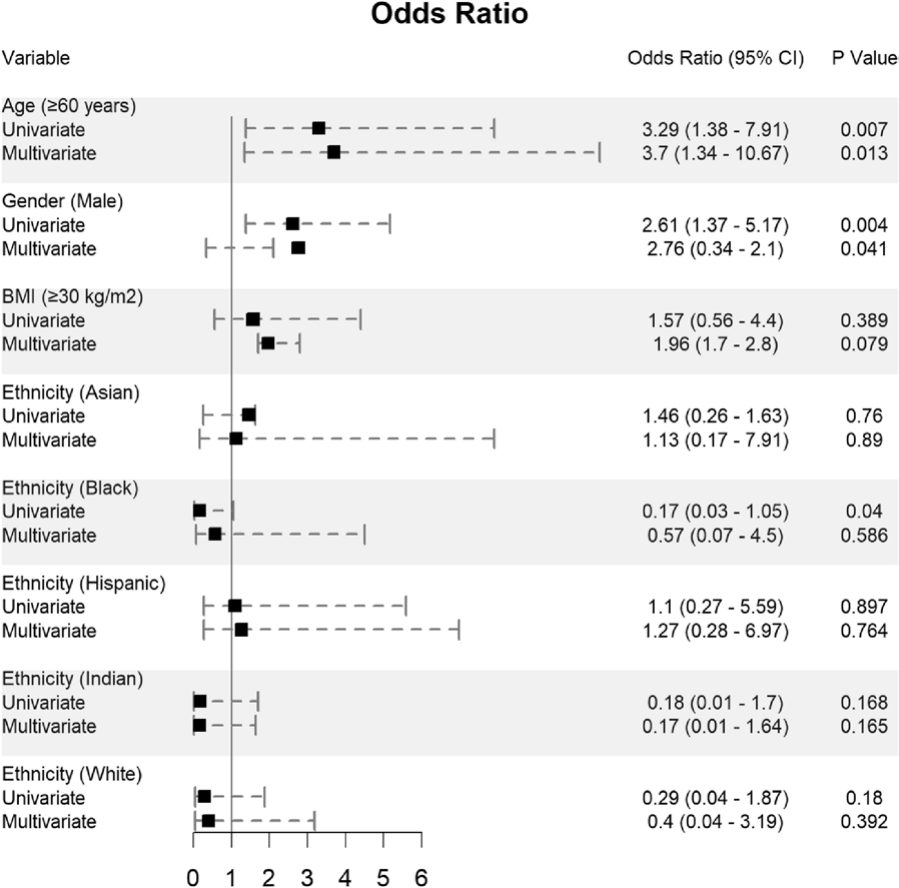
Predictors of mortality in patients hospitalized for DKA and SARS-CoV-2 (n = 243)

**Fig. 3:**
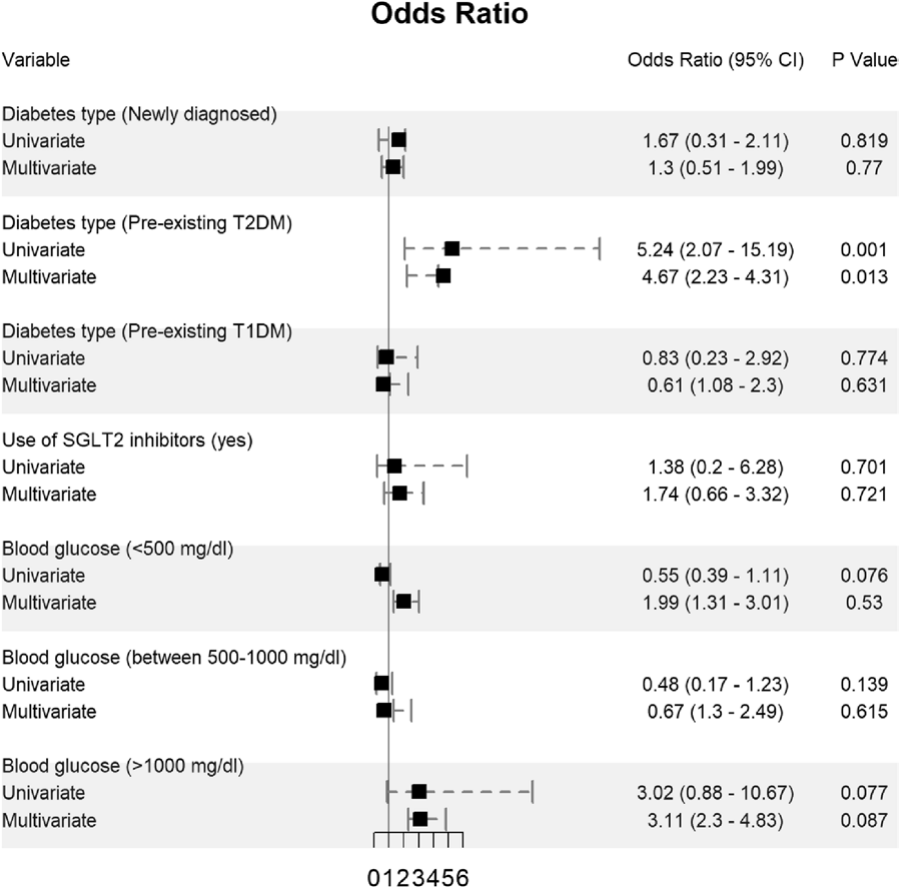
Predictors of mortality in patients hospitalized for DKA and SARS-CoV-2 (n = 243)

**Fig. 4:**
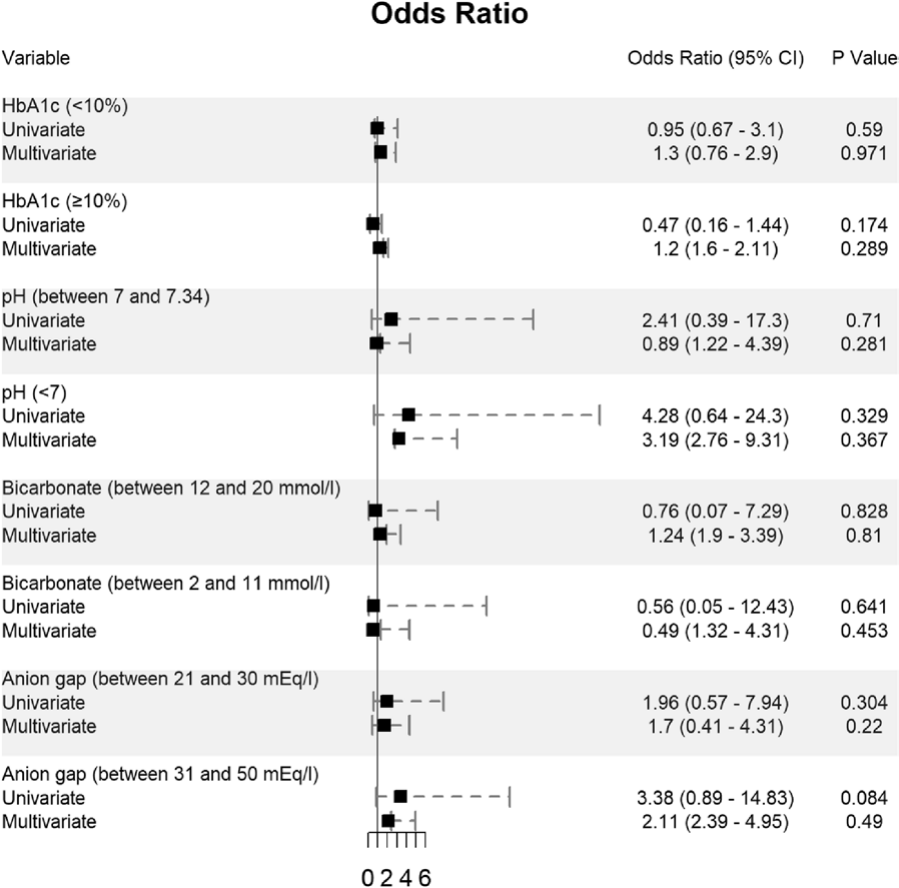
Predictors of mortality in patients hospitalized for DKA and SARS-CoV-2 (n = 243)

Potential determining variables associated in survival and death groups were analyzed through binary logistic regression analysis and shown in Fig. 2, Fig. 3 and Fig. 4. As expected, old age [≥ 60 years] (OR 3.29, 95% CI 1.38–7.91; *p* = 0.007), male gender (OR 2.61, 95% CI 1.37–5.17; *p* = 0.004), and BMI ≥ 30 kg/m^2^ (OR 1.57, 95% CI 0.56–4.4; *p* = 0.389) are associated with increased odd ratio for death; Fig. 2. Among the diabetes types, patients who presented with pre-existing diabetes mellitus type 2 had a very high OR of dying (OR 5.24, 95% CI 2.07–15.19; *p* = 0.001). In opposite, patients with pre-existing diabetes mellitus type 1 had a much lower OR of 0.83 for mortality (95% CI 0.23–2.92); Fig. 3. Other predictors for increased risk of succumbing included blood glucose level ≥ 1000 mg/dl (OR 3.02, 95% CI 0.88–10.67), low pH of < 7 (OR 4.28, 95% CI 0.64–24.3), and high anion gap [between 31 and 50 mEq/l] (OR 3.38, 95% CI 0.89–14.83); Fig. 3 and Fig. 4.

These variables were considered needing further evaluation and, thus, were included in multivariate regression analysis. Nevertheless, multivariate analysis confirmed old age [≥ 60 years], male gender, and a pre-existing diabetes mellitus type 2 were significantly associated with increased death. Although univariate analysis showed black ethnicity was significantly associated with increased mortality (*p* = 0.04), however, this finding was not reciprocated by multivariate analysis; Fig. 2.

## Discussion

This is the largest meta-analysis on the development of DKA in patients with SARS-CoV-2. This study involving 639 patients from 68 observational studies found majority of the DKA patients diagnosed with SARS-CoV-2 were adults (52.3%), men (58.4%) and had pre-existing type 2 diabetes mellitus (48.3%).

DKA is one of the most common and serious hyperglycaemic emergency; and is considered a precipitating event that frequently occurs due to infection [often pneumonia or urinary tract infection], and discontinuation of or inadequate insulin therapy [76, 77]. Adults of any age may develop severe SARS-CoV-2 and experience adverse outcomes, especially those with comorbidities [78, 79]. Most children with SARS-CoV-2 have mild symptoms or have no symptoms at all [80], however, adults are at higher risk to experience more severe COVID-19 infection than children [81]. Factors proposed to explain the difference in severity of COVID-19 in children and adults include: 1- age-related increase in endothelial damage and changes in clotting function; 2- higher density, increased affinity and different distribution of angiotensin converting enzyme 2 receptors and transmembrane serine protease 2; 3- pre-existing coronavirus antibodies (including antibody-dependent enhancement) and T cells; 4- immunosenescence and inflammaging, including the effects of chronic cytomegalovirus infection; 5- a higher prevalence of comorbidities associated with severe COVID-19 and 6- lower levels of vitamin D [82]. Hence, lower rate of children SARS-CoV-2 patients with DKA in our review can be justified by the fact that the high severity of COVID-19 tends to be much lower in children compared to adults.

DKA is thought to happen most often in patients with diabetes mellitus type 1 [49, 83], however, this conceptualization is not true and we report fourfold higher rate of DKA in the diabetes mellitus type 2 patients compared to diabetes mellitus type 1. Type 2 diabetes mellitus patients have high susceptibility to DKA under stressful conditions such as trauma, surgery or infections [83]; and majority of the DKA cases worldwide occur in patients with type 2 diabetes due to its higher prevalence [84, 85]. DKA occurs more commonly in adult COVID-19 patients with type 2 diabetes mellitus mainly because the worldwide prevalence of diabetes mellitus type 2 is estimated at 9.3 percent in adults, equivalent to 463 million people [86]. Type 2 diabetes accounts for over 90 percent of patients with diabetes [86, 87].

In our review, males gender predominated development of DKA in SARS-CoV-2 patients, a finding suggested in most of the reports [11–13, 15–17, 19, 20, 23, 25, 26, 31, 33, 36, 37, 39, 47, 50, 52, 55–58, 60–64, 69, 74, 75] and in contradiction with data from other reports suggesting an equal proportion of DKA cases in COVID-19 patients for both genders [30, 34, 38, 49]. Lifestyle, body fat distribution, hormonal factors, susceptibility to glucotoxicity and lipotoxicity, and changes in insulin sensitivity have been described as potential factors of DKA and possible mechanisms of male predominance [88]. However, male excess in DKA in our review might be attributed mainly to the differences in the inclusion criteria and the population age groups included in the studies; or can be explained by social factors as women are often the primary caregivers for their families, assuming the responsibility of family members’ disease management, at the expense of their own treatment [89].

A comparison of the current results with findings from previous studies can offer some validation of the findings of this present meta-analysis and identify methodological differences in their approaches. Regarding the mortality rate in patients who developed DKA during SARS-CoV-2 infection, we report an overall similar and slightly lower death rate [25.9%] compared to the previous two systematic meta-analyses [28.9 and 29%, respectively] [90, 91]. The current meta-analysis is more comprehensive and included a total of 68 studies [8–75] including a total of 639 patients; whose details on final treatment outcome were available; in comparison to smaller sample size in previous meta-analyses [sample size: n = 45 and n = 21, respectively] [90, 91]. The inclusion of 48 recently published studies [8–14, 16, 21, 23–26, 28, 30, 32–35, 40, 42–46, 48, 50, 51, 53, 54, 56–59, 61–64, 66–75] contributed to the refinement on evidence of the demographic, biochemical, and clinical characteristics; in addition to final therapy outcome in COVID-19 patients with DKA.

Consistent with previous meta-analysis, we found development of DKA in SARS-CoV-2 patients was highest in the Hispanics and blacks (24.4% and 15.3%, respectively) [91]. Moreover, we found mortality rate in DKA patients infected with COVID-19 was significantly very high in patients with Hispanic ethnicity [27%, *p* = 0.001] in whom risk of acquiring SARS-CoV-2 and clinical prognosis of this viral infection was previously described as high and poor [92, 93]. Because most of the studies included in our review that reported the ethnicity of DKA cases infected with COVID-19 were either from the United States of America, India or United Kingdom; representation of other ethnicities at risk to develop DKA during COVID-19 can be misleading. For instance, we report a low prevalence of DKA in Asian population, yet, a systematic review and meta-analysis reported the highest DKA incidence rates in Chinese people [94].

In line with our findings, severely low pH (i.e. pH of < 7) has been identified as an important predictor of mortality in patients with DKA and COVID-19 compared to those who survived (*p* = 0.047) [90, 91]. Very high uncontrolled random blood glucose level (> 1000 mg/dl) was the other biochemical parameter at presentation that differed significantly between the survival and death groups in DKA patients infected with SARS-CoV-2 (*p* = 0.048); a finding suggested in previous studies [91, 95, 96] and in contradiction with data from case reports demonstrating death in DKA cases during COVID-19 infection when their blood glucose levels were kept at < 500 mg/dl [27, 59, 63]. Moreover, increasing age in combination with male gender and BMI ≥ 30 might denote seriously sick patients who can potentially have more morbidity and propensity to die. The majority of patients hospitalized with SARS-CoV-2 are older and seemed to have underlying medical conditions [97, 98], with increased age being associated with clinical severity, including case fatality [97, 99]. Fortunately, however, mortality from DKA in elderly people have also declined dramatically during the past 10 years [100]. Therefore, these patients should be identified at the earliest and treated preferably in a special care set up to avoid morbidity and mortality. It is worth mentioning increasing age in patients may result in increased hospital stay and might put SARS-CoV-2 patients at risk to develop medical complications like coagulopathy, pneumonia, acute respiratory distress syndrome, organ failure and nosocomial coinfections [97, 101]. The presence of these factors in severely ill patients may have necessitated the use of advanced therapies like renal replacement therapy or ventilator support which would have delayed hospital discharge [102]. Although COVID-19 has a higher survival rate than other chronic diseases, the incidence of complications in the geriatric population are considerably high, with more systemic complications [103]. Of the patients admitted to hospital for management of COVID-19, 49.7% (36,367 of 73,197) had at least one complication [104]. Overall, complications and worse functional outcomes in patients admitted to hospital with SARS-CoV-2 are high in old people, and even in young, previously healthy individuals; and COVID-19 complications could strain health system for years.

In our review, the odd ratio of mortality was the highest in DKA patients with the pre-existing the diabetes type 2 variable [OR 5.24, 95% CI 2.07–15.19; *p* = 0.001]; and DKA patients with pre-existing type 1 diabetes had very low OR of death [OR 0.83, 95% CI 0.23–2.92; *p* = 0.774]. In diabetes mellitus type 2 diabetes, underlying severe illness is almost always the direct cause of both the DKA and ensuing death; while in diabetes mellitus type 1 diabetes, DKA is most often caused by missed insulin doses but death is rare with prompt treatment [49].

There is growing evidence to suggest that SARS-CoV-2 might cause diabetes in some people [105, 106]. In our study, out of the 639 DKA patients infected with SARS-CoV-2, there was (n = 75, 11.7%) newly diagnosed diabetes mellitus cases at admission. Of these 75 cases, 22 had HbA1c > 9.0% (ranging from 9.5% to 17.4%) [8, 9, 11, 12, 20, 22, 33–35, 39, 40, 44, 45, 53, 56, 67, 69] and three of which had a BMI > 30 [44, 69], suggesting these patients had undiagnosed diabetes mellitus and improbable was caused by SARS-CoV-2 infection. DKA in COVID-19 patients was the least to occur in newly diagnosed diabetes cases probably as a result of increased diabetes screening and early recognition, DKA now occurs more frequently in persons with established diabetes rather than at the time of the initial diagnosis [100]. COVID-19 likely unmasked existing diabetes mellitus by aggravating its metabolic complications rather than causing the new-onset diabetes in these patients.

Out of the 11 (1.7%) DKA cases infected with COVID-19 who were taking SGLT2 inhibitors, two patient [18.2%] were diagnosed with SGLT2-associated euglycemic DKA [blood glucose < 250 mg/dl at presentation] [16, 48]; in addition to seven patients who had gestational diabetes mellitus [53, 65, 70]. Euglycemic DKA is a rare life-threatening complication associated with the use of SGLT2 inhibitors in patients with type 2 diabetes that may be unnoticed, particularly in COVID-19 pandemic, due to the absence of significant hyperglycaemia, delaying its treatment [16]. Given their undisputed cardiovascular and renal benefits, these medications are common in patients with type 2 diabetes [107]. There are recommendations that patients using SGLT2 inhibitors should be monitored for ketosis using available home testing kits in case of infections and should discontinue the medication in case of SARS-CoV-2 while the administration of insulin is considered the safest pharmacotherapy choice [108].

### Limitations

First, while most of the evidence discussed were based on few cohorts, some case series and many case reports, many of these are small and not necessarily generalizable to the current COVID-19 clinical environment. Second, to asses factors associated with mortality, larger cohort of patients is needed. Third, almost all studies included in this review were retrospective in design which could have introduced potential reporting bias due to reliance on clinical case records. Fourth, study was not registered in Prospero, an international prospective register of systematic reviews, as this might have added extra work and the merit was mostly limited to the avoidance of duplication. Last, the study population included paediatric patients and hence its results cannot be generalized to adult patients.

## Conclusion

Patients with diabetes are at increased risk of severe complications from SARS-CoV-2 which may include DKA. Acute diabetes-related DKA in SARS-CoV-2 patients lead to increased mortality; key determinants are individuals with pre-existing diabetes mellitus type 2, older age [≥ 60 years old], male gender, BMI ≥ 30, blood glucose level > 1000 mg/dl, and anion gap ≥ 30 mEq/l.

## Supplementary Information

**Additional file 1.** Search terms.

**Additional file 2.** Search outcomes of all studies found via electronic search databases.

## Data Availability

Data are available upon request, please contact author for data requests.

## Abbreviations

COVID-19: Coronavirus disease 2019
DKA: Diabetic ketoacidosis
NOS: Newcastle–Ottawa scale
PRISMA: Preferred Reporting Items for systematic reviews and meta-Analyses
SARS-CoV-2: Severe acute respiratory syndrome coronavirus 2
SGLT2: Sodium-glucose Cotransporter-2 inhibitors
