# Supplementary material for: Diabetic ketoacidosis in patients with SARS-CoV-2: a systematic review and meta-analysis
Source: Diabetol Metab Syndr. 2021 Oct 26;13:120. doi: 10.1186/s13098-021-00740-6 (PMC8547563; doi:10.1186/s13098-021-00740-6)
Supplement: Supplementary file 1 — Additional file 1. Search terms. [file 13098_2021_740_MOESM1_ESM.docx]

**Additional File 2: Search terms**

Electronic databases (Proquest, Medline, Embase, Pubmed, CINAHL, Wiley online library, Scopus and Nature) were searched from 1 December 2019 to 30 June 2021 in the English language using the following keywords alone or in combination: *COVID-19* OR *SARS-CoV-2* AND *diabetic ketoacidosis* OR *DKA* OR *ketosis* OR *ketonemia* OR *hyperglycaemic* *emergency* OR *hyperglycaemic* *crisis*.
